# Show Me What You Have Inside—The Complex Interplay between SIBO and Multiple Medical Conditions—A Systematic Review

**DOI:** 10.3390/nu15010090

**Published:** 2022-12-24

**Authors:** Natalia Sroka, Alicja Rydzewska-Rosołowska, Katarzyna Kakareko, Mariusz Rosołowski, Irena Głowińska, Tomasz Hryszko

**Affiliations:** 1 2nd Department of Nephrology and Hypertension with Dialysis Unit, Medical University of Białystok, 15-276 Białystok, Poland; 2 Department of Internal Medicine and Hypertension, Medical University of Białystok, 15-540 Białystok, Poland

**Keywords:** SIBO, gut microbiota, dysbiosis, LPS, breath test, rifaximin

## Abstract

The microbiota, as a complex of microorganisms in a particular ecosystem, is part of the wider term—microbiome, which is defined as the set of all genetic content in the microbial community. Imbalanced gut microbiota has a great impact on the homeostasis of the organism. Dysbiosis, as a disturbance in bacterial balance, might trigger or exacerbate the course of different pathologies. Small intestinal bacterial overgrowth (SIBO) is a disorder characterized by differences in quantity, quality, and location of the small intestine microbiota. SIBO underlies symptoms associated with functional gastrointestinal disorders (FGD) as well as may alter the presentation of chronic diseases such as heart failure, diabetes, etc. In recent years there has been growing interest in the influence of SIBO and its impact on the whole human body as well as individual systems. Therefore, we aimed to investigate the co-existence of SIBO with different medical conditions. The PubMed database was searched up to July 2022 and we found 580 original studies; inclusion and exclusion criteria let us identify 112 eligible articles, which are quoted in this paper. The present SIBO diagnostic methods could be divided into two groups—invasive, the gold standard—small intestine aspirate culture, and non-invasive, breath tests (BT). Over the years scientists have explored SIBO and its associations with other diseases. Its role has been confirmed not only in gastroenterology but also in cardiology, endocrinology, neurology, rheumatology, and nephrology. Antibiotic therapy could reduce SIBO occurrence resulting not only in the relief of FGD symptoms but also manifestations of comorbid diseases. Although more research is needed, the link between SIBO and other diseases is an important pathway for scientists to follow.

## 1. Introduction

The term microbiome is generally used to define the set of all genetic material of a microbial community. On the other hand, the microbiota is commonly described as a complex of microorganisms in a particular environment. The human gut microbiota inholds above 1500 species spread in more than 50 phylas [1], but it is dominated mainly by two of them—Bacteroidetes and Firmicutes [2]. The intestinal bacteria have a multidirectional impact on individual organs and the whole body by the synthesis of diverse metabolic products or supporting prevention from the invasion of pathogens by colonizing mucosal surface [3]. Bacterial equilibrium is crucial in the maintenance of immunity and homeostasis of the organism. Dysbiosis, as a disturbance in bacterial balance, might trigger or exacerbate the course of different pathologies.

Small intestinal bacterial overgrowth (SIBO) is a condition defined by the number of colonic-type bacteria in the small gut equal or greater than $10^{5}$ colony forming units per milliliter (CFU/mL) as well as an alteration in the standard ratio of small bowel microbiota [4]. Common complaints in patients with SIBO are general manifestations and gastrointestinal (GI) symptoms like abdominal pain or defecation rhythm disturbances. Higher permeability for lipopolysaccharide (LPS) is mentioned as the main cause of underlying pathology, which leads to stimulation of inflammatory response and chronic proinflammatory status. Conditions concomitant with SIBO may predispose to the development of intestinal dysbiosis itself. Disorders in the structure and functionality of the intestinal wall as a result of disease or its treatment, higher than normal concentrations of substances like ghrelin, leptin, TMAO (trimethylamine-N-oxide), or the presence of pro-inflammatory cytokines could take part in the induction and development of SIBO.

The jejunal aspirate is known as the gold standard for diagnosing SIBO. However, breath tests (BTs) with glucose or lactulose (glucose breath test [GBT] or lactulose breath test [LBT] respectively) are used more often, because of their non-invasiveness [5].

In recent years there has been growing interest in the influence of gut microbiota, especially SIBO, and its impact on the whole human body as well as individual systems. The objective of our research was thus to investigate and systematize the relationships mentioned above. To better understand these relations, we performed a review of the literature.

## 2. Materials and Methods

For the identification of relevant publications, a standardized search strategy was used. The PubMed database was searched up to July 2022 with the terms “SIBO”, “Small Intestinal Bacterial Overgrowth”, “gut microbiota”, “gut bacteria”, “microbiome”, “microbiota”, “dysbiosis”, “breath test”, “gut axis” in various combinations with the terms “related”, “altered”, “treatment”, and “disease”. Based on these results we could determine which body systems to focus on. Subsequently, we broadened our trawl by linking the term “SIBO” with specific organs or diseases: “H.pylori infection”, “IBS”, “IBD”, “Crohn’s disease”, “Colitis ulcerosa”, “celiac disease”, “obesity”, “abdominal surgery”, “NAFLD”, “cirrhosis”, “gallstone”, “pancreas”, “pancreatitis”, “cystic fibrosis”, “heart failure”, “atherosclerosis”, “coronary artery disease”, “DVT”, “diabetes”, “thyroid”, “hypothyroidism”, “hyperthyroidism”, “Parkinson”, “brain fogginess”, “systemic sclerosis”, “rosacea”, “kidney”, “CKD”, “AKI” and “IgA nephropathy”. Additional publications were retrieved from citations of manuscripts found in the above search.

## 3. Results

**Figure 1:**
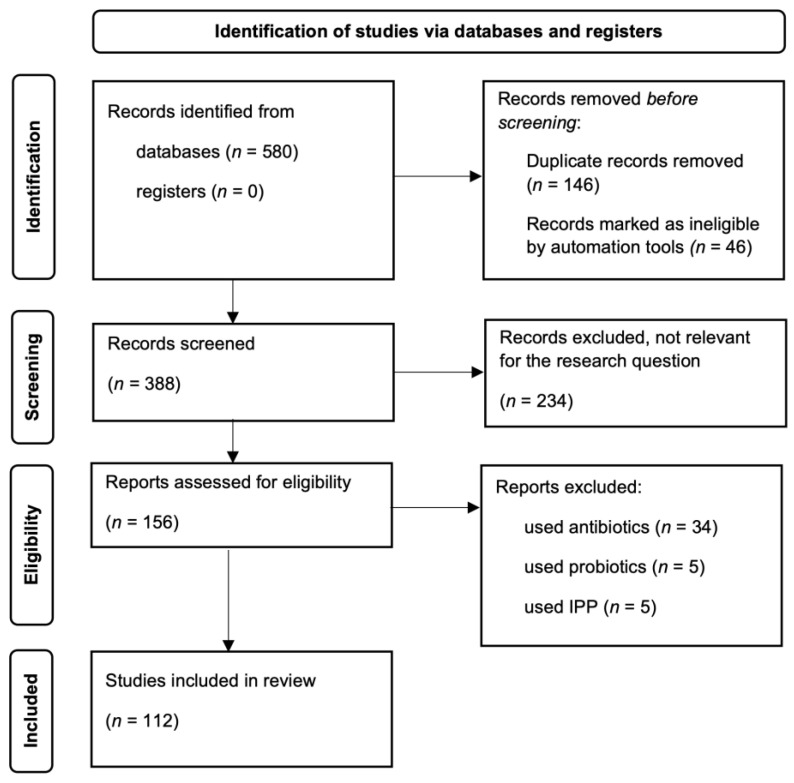
Search strategy and results.

The search strategy lets us identify 580 articles. Publications were included if they assessed SIBO in humans. Studies were limited to original articles available in the English language. Dual papers and titles not relevant to the research question were excluded. Review articles, metanalyses, meeting abstracts, case reports, editorials, and commentaries were excluded as well. We also excluded articles, when the main test group had taken: (a) antibiotics, (b) probiotics, (c) IPP. Finally, we identified 112 eligible articles, which are included in this paper. A schematic PRISMA flow diagram for the selection of the studies included in this review is presented in Figure 1. Additionally, quoted articles were categorized and summarized in Table 1.

## 4. Discussion

### 4.1. Definition and Pathophysiology

Small intestinal bacterial overgrowth is a disorder characterized by differences in quantity, quality, and location of the small intestine microbiota [5]. The excess bacterial population in the small bowel (the standard definition is $10^{5}\;$CFU/mL of proximal jejunal aspiration) is accompanied by the appearance of Gram-positive and Gram-negative aerobic organisms as well as anaerobes, which are usually more common in the colon [118,119]. The main bacteria associated with SIBO include Streptococcus, Staphylococcus, Bacteroides, Lactobacillus, and Enterobacteriaceae–Escherichia, Klebsiella, or Proteus [120].

Patients suffering from SIBO usually complain of general symptoms like weight loss, weakness, and other manifestations specific to the gastrointestinal tract—abdominal pain, bloating, diarrhea, constipation, and belching. Disturbances in the gut lumen are induced by bacterial overgrowth, which entails nutritional deficiencies. Increasing malabsorption exacerbates the condition and leads to more severe complications like anemia, fat-soluble vitamin deficiencies, or hypoproteinemia [5]. Thereupon SIBO could be diagnosed in symptomatic patients when proximal jejunal aspirate contains >$10^{3}\;$CFU/mL [121].

The digestive system possesses different mechanisms preventing the excess of small intestine microbiota. Many factors influence the development of SIBO: gastrointestinal motility, gastric acid and pancreatobiliary secretion, anatomy of the tract, and the immunological system. The most important of these—motility, includes stomach emptying as well as peristaltic movements in the small bowel which may result in the delayed transition of bacteria to the colon. Gastric acid, as the first guarding mechanism, limits the quantity of bacteria in the gut, by creating a hostile environment for bacterial population growth [5,119]. The other protector is the ileocecal valve—its lower pressure is highly associated with SIBO occurrence [122].

### 4.2. Diagnosis

Initially, SIBO used to be diagnosed together with other abnormalities of the GI tract, including postsurgical changes. Nowadays, mostly due to its non-specificity and often asymptomatic course, a symptom-based diagnosis encounters some difficulties. Thus, it was crucial to find a proper diagnostic technique. The present methods could be divided into two groups—invasive, including the gold standard—small intestine aspirate culture and non-invasive, breath tests [4].

The number of aerobic and anaerobic bacteria per milliliter of jejunal aspirate equal to or greater than $10^{5}$ suggests SIBO [123]. According to The North American Consensus on Breath Testing it is advised to apply lactulose or glucose pending BT; an increase of either hydrogen level 20 p.p.m. from baseline by 90 min or methane level 10 p.p.m on BT for both carbohydrates is considered positive [121].

Although endoscopy, due to its directness, is acknowledged as the gold standard for diagnosing SIBO, it carries several disadvantages [4]. Besides performing the actual procedure, significant other problems emerge: high cost, the additional risk for the patient, improper sample collection leading to contamination by oropharyngeal flora, and lack of transparent cutoff determining a positive aspirate. On the other hand, breath tests are characterized by lower sensitivity (52–63% for GBT, 31–68% for LBT) and specificity (82–86% for GBT, 44–100% for LBT) [5]. Subjects are advised to prepare correctly for the examination which creates the greatest impediment. It is important to avoid antibiotics for four weeks before BT and promotility medications as well as laxatives one week before. Consuming complex carbohydrates is forbidden on a preceding day, fasting time should be about 8 to 12 h and smoking is not allowed on the day of BT. Pro- and prebiotics as well as proton pump inhibitors (PPI) are not limited, but the latter is not recommended on the test day [121]. Most reliable results are obtained when patients abide by all those rules.

Interestingly, not only BT or jejunal aspirate could be considered to investigate overgrowth in the small intestine. Increased fecal calprotectin concentration was found in subjects with systemic sclerosis and was connected with SIBO occurrence. Fecal calprotectin may be considered as a prospective, non-invasive test in SIBO diagnostics [111].

### 4.3. Prevalence in Other Conditions

SIBO could be the only diagnosis in a patient, but it frequently accompanies or is accompanied by other conditions. The first connection was other disorders of the gastrointestinal system, subsequently, the relationship between SIBO and other systemic diseases was considered.

#### 4.3.1. Gastroenterology

The digestive system is highly exposed to many microorganisms; hence it evolved complex defense mechanisms. Dysfunction of protective barriers may lead to the development of SIBO. Hypochlorhydria, as a result of a decrease of gastric juice in the course of aging, abuse of PPI [124,125], or colonization with Gram-negative Helicobacter pylori, are well-known risk factors for excess of gut bacteria [119,126]. A rise in the gastric pH yields dysbiosis in the stomach and small bowel [127]. Moreover, Enko and Kriegshäuser observed that patients with H. pylori infection and positive 13C-urea breath test (13C-UBT) have an even higher risk of SIBO [6].

The occurrence of many bowel diseases correlates significantly with the quantity and quality of small intestine flora. Manifestations similar to SIBO are observed in irritable bowel syndrome (IBS) multifactorial disease, therefore initially both were associated together. An activation of the immune system and expansion of inflammatory bodies in intestinal mucosa [128] and lamina propria [129] are observed in a course of IBS. These mechanisms may implicate the mucosal wall and increase intestinal permeability [10,13]. On the other hand, the presence of SIBO triggers immune activation as well [130]. Therefore, there are plenty of studies describing the co-occurrence of IBS and SIBO [7,8,11,12,15,17,19]. However, there is a big problem with the selection of a suitable method of diagnosis between breath tests and jejunal aspirate. LBT seems to create the most discrepancies [9,16,20] but combined with scintigraphic orocecal transit could achieve actual results [20]. Chronic diarrhea, constipation, abdominal pain, or bloating are present without a tangible cause. It was discovered that SIBO appeared less often in IBS-constipation subtype than in those with diarrhea [18]. In many patients, remission or reduction of symptoms after therapy with rifaximin was demonstrated, which may indicate SIBO as a causative factor of IBS [7,14,131]. The SIBO-IBS hypothesis is an interesting one and warrants more investigation.

Consequently, scientists took a closer look at inflammatory bowel diseases (IBD). The more frequent prevalence of SIBO with Crohn’s disease (CD) than ulcerative colitis (UC) was observed in the majority of research [21,24,25,28,29,30,31]. Simple explanations could include easier access to the small bowel (mainly in CD), frequent fistulas and strictures, and sometimes required remedial surgical procedures even with resection of the ileocecal valve, which have a serious impact on intestinal motility [23]. On the other hand, the elevation of pro-inflammatory cytokines (IL-6, IL-8, TNF-alpha) and anti-inflammatory cytokine (IL-10) is observed in UC. Intestinal inflammation might damage enteric nerves or smooth muscles which leads to altered GI motility [26]. Both mechanisms are responsible for the prolongation of orocecal transit time (OCTT) and increased SIBO incidence in patients with IBD [22,26,28]. Interestingly, activity scores of Crohn’s disease, as well as ulcerative colitis, could be diminished by rifaximin treatment which significantly improves patients’ quality of life [132,133]. Additionally, Yang et al. affirmed better clinical efficacy of mesalazine after eradication of SIBO with rifaximin [27].

Celiac disease seems to certainly correlate with intestinal microbiota composition. The connection between SIBO and celiac disease has been studied since 1970 and the prevalence of SIBO was as high as 66.66% according to published data [32,33,34,35,38]. Precursory studies implied that due to GI dysmotility, the patient’s condition was further complicated by the presence of gut dysbiosis. Cholecystokinin is a hormone responsible for stimulations of intestinal motility and its downregulation in the celiac population is mentioned as one of the causes of dysmotility [134]. Moreover, some studies showed in these patients greater concentration of neurotensin—an inhibitor of upper GI peristalsis [135]. Additionally, intraepithelial lymphocytes are mediators of mucosal damage in SIBO and celiac disease, which implies a role of gut bacteria in the pathogenesis [136]. Eradication of SIBO with rifaximin yielded remission of symptoms in patients that had not responded to gluten-free diet [33]. Surprisingly, further studies showed no response to antibiotic therapy and a lower prevalence of SIBO in celiac disease, which was confirmed by aspirates of bacterial culture from small bowel–the gold standard of diagnosis [36,37]. Contrary to expectations, both conditions might exist independently.

Abdominal or pelvic surgery is also considered a risk factor for excess of gut bacteria [39,42,43]. Gastrectomy entails a decrease in gastric acid production [42], while cholecystectomy affects biliary secretion. Moreover, several ventral surgeries could induce disorders in GI structure and motility. Additionally, Kim et al. observed dependence between the type of the procedure and an increase in H2 concentration during BT, which was the highest after gastrectomy [42]. Similarly to other conditions, oral rifaximin diminishes gastrointestinal complaints suggesting a role of gut dysbiosis in the pathogenesis of symptoms [40,43]. Nowadays, as bariatric procedures become the crucial treatment of obesity, and its use becomes widespread, data show disturbed intestinal motility in those patients [41,44]. Approximately 80% of individuals after bariatric surgeries suffer from symptomatic SIBO [44] and the highest proportion is found after Roux-en-Y gastric bypass [41]. It might be associated with greater weight loss than in other bariatric procedures [41].

However, obesity in a multifactorial way can also be a cause of SIBO. First of all, abnormal levels of leptin and ghrelin were observed in obese people, which fosters dysmotility [46]. Secondly, cluster contractions of myenteron as a result of the increase of intestinal Migrate Motor Complex (MMC), typical for small bowel obstruction and common in advanced liver cirrhosis, were noticed as well [46,47,48]. On the other hand, the difference in the composition of excess gut microbiota between obese and non-obese patients might influence the function of the intestinal mucosa (decline in the proportion of Bacteroides to Firmicutes). It leads to increased calorie intake, accumulation of fat in the adipocytes by greater production of short-chain fatty acids, and rise of ratio of visceral to subcutaneous fat area [47], as well as an increase of gut permeability for LPS and endotoxins, what leads to chronic proinflammatory status. This vicious circle is observed in SIBO and obesity as a constellation of different relationships. Additionally, a higher prevalence of intestinal dysbiosis in people suffering from metabolic syndrome was established during studies on SIBO and adiposity [47].

Solid organ dysfunction might also have an impact on the prevalence of dysbiosis in the small gut, mainly by affecting GI motility [62]. Non-alcoholic fatty liver disease (NAFLD), which could transform into non-alcoholic steatohepatitis (NASH), end-stage of liver dysfunction, or even hepatocarcinoma is the most widespread liver disorder in developed Western countries [137]. Obesity, as well as metabolic syndrome and type 2 diabetes mellitus, are the main risk factors for NAFLD [138]. Hence, similar implications of intestinal dysbiosis as mentioned above were noticed. A lower concentration of Bacteroides (which is lacking genes responsible for choline metabolism) was noticed in NASH patients irrespectively of BMI and fat intake [139]. Moreover, disorders of choline metabolism due to an increase of trimethylamine-N-oxide (TMAO) in the systemic circulation, lead to imbalanced lipid homeostasis [140]. Furthermore, several studies report a permanent elevation of the concentration of endogenous ethanol, which could be explained by increased fermentation in the gut lumen [141]. All those factors might be implicated in the development of NAFLD. A high prevalence of SIBO is observed in NAFLD population [49,50,51,52,53,54,55,56]. SIBO, by inducing an immunological fluctuation, could lead to chronic inflammation, mitochondrial disorders, a cumulation of lipids in hepatocytes, and NASH. However, most of the recent evidence is based on animal subjects or small human studies, so the relationship between gut microbiota and NAFLD is still more hypothesized than established.

SIBO is frequently diagnosed in liver cirrhosis [61,62,63,67] and it is associated with its severity [64,67], like the occurrence of hepatic encephalopathy or ascites and the Child-Turcotte-Pugh class. In various studies, the prevalence of SIBO is greater in patients with Child’s C than Child’s A cirrhosis [57,58,59,60,64]. Intestinal permeability and immunodeficiency predispose to a proinflammatory condition even without an obvious source of infection. Thereupon, the risk of development of spontaneous bacterial peritonitis is greater in the presence of SIBO in cirrhotic patients [65,66]. Moreover, ascites and increased serum bilirubin concentration could forecast the presence of SIBO [64].

Additionally, parenchymatous liver dysfunction is a risk factor for gallstone disease. Until present, only a few studies have been published, that compare it with SIBO. Both disorders are characterized by similar manifestations, like abdominal discomfort, nausea, belching, bloating, or fullness in the stomach after eating. Kim et al. observed a higher concentration of exhaled hydrogen and a prevalence of SIBO in subjects with gallstones [69]. The primary cause is speculated to be the result of dysfunction of gallbladder mucosa and the decreased secretion and antibacterial impact of bile in the GI tract due to excessive numbers of intestinal microbiota. On the other hand, one of the bile acids–deoxycholate—is produced from cholate in the gut lumen by gram-positive anaerobes. Prolonged OCTT entails a rise of deoxycholate absorption and imbalanced composition of bile, which becomes more lithogenic [68,69]. However recent studies have not yet explained the link between increased OCTT and serum bile acids.

Another organ potentially influenced by intestinal bacterial disorders is the pancreas. Chronic pancreatitis (CP) is a worldwide multifactorial disease with a high mortality [142]. Similar manifestations of CP and SIBO, like abdominal pain, diarrhea, or bloating suggest a connection between both. The studies revealed a higher prevalence of excessive numbers of bowel microbiota in CP patients than in healthy ones, regardless of etiology and it was associated with the Mayo Score [74,76]. Diabetes mellitus [75] and pancreatic exocrine insufficiency could also contribute to the progress of SIBO. Several mechanisms are proposed. Firstly, decreased secretion of the pancreatic juice, which has antimicrobial functions as well, could promote the overgrowth of gut bacteria and malabsorption [73]. Furthermore, observed prolonged OCTT could be evoked by diabetes neuropathy, opioid intake, or maldigestion. Pancreatic enzyme replacement therapy (PERT) is crucial in CP treatment. Interestingly, the persistence of abdominal symptoms after PERT suggests SIBO as the etiology of exacerbation of the disease [73]. Frequently the condition is improved after empiric therapy with rifaximin [71,73]. However, all authors highlight the heterogeneity and the small number of test groups, which might have affected the results. Moreover, also a discrepancy between glucose and lactulose BTs exists. The latter shows a higher number of SIBO diagnoses in the CP group, probably caused by faster lactulose transit to the colon than expected and most results are considered as false positive [143], therefore glucose BT is preferable [70].

Studies also exist linking acute pancreatitis to gut microbiota. Although the duration of manifestations is not long, even 24 h is enough to initiate disturbances in the gut homeostasis [144]. The main reasons appear to be GI tract hypomotility with higher bowel permeability as well as decreased exocrine function and carbohydrates digestion due to acute pancreatitis (AP). A hospitable environment for the expansion of microbiota is created by these mechanisms [77,78]. The consequence is endotoxemia which yields a higher risk of complications like necrosis or secondary infection of the pancreas and mortality. The severity of AP correlates with the prevalence of SIBO [77].

Cystic fibrosis (CF) is a genetic disorder, which significantly affects the digestive system. Mutation of the CFTR gene leads to decreased secretion of fluid and bicarbonate from epithelium and accumulation of abnormal, concentrated mucus in the intestinal lumen. Thickened mucus is responsible for prolonged OCTT. Moreover, damage of Paneth cells, present in CF, affects small intestine motility as well [79,145]. Pancreas dysfunction and all factors mentioned above predispose to an excessive number of intestinal microbiota. The prevalence of SIBO in CF approaches 30% to 50% [145] and it might exacerbate gastrointestinal symptoms in CF. Because of similar manifestations, it is difficult to discern between both conditions. A huge bacterial burden may induce mucus secretion and impair its function. Empiric antibiotic treatment with rifaximin or metronidazole in CF results in improved digestion and absorption of nutrients as well as weight gain and appetite improvement [80,81]. Probiotic therapy may also amend the quality of life in the CF population by positive impact not only on GI but also on respiratory function by reducing pulmonary inflammation [80,81,146]. Unfortunately, the majority of studies were performed on animals and further research is crucial to understand and establish the dependence between SIBO and CF.

#### 4.3.2. Cardiology

The gastrointestinal tract may seem, at first glance, to be the only target of SIBO signs and symptoms but scientists have explored its connections with other organs and systems. One of them was the cardiovascular system. Heart failure (HF) has considerable effects on the gut bacteria [147]. Co-existence of both entities, disturbances of the gut microbiota equilibrium as well as prevalence and severity of HF were examined in a lot of studies [82,148]. Ischemia of the small intestine wall can be caused by a decreased output of the heart [148,149]. Consequently, it may lead to bowel dysfunction, and higher intestinal permeability, and correlate with the severity, complications, and progression of HF [82,147,149,150]. The possible trigger might be lipopolysaccharide (LPS)–one of the components of Gram-negative bacteria, classified as a proinflammatory factor,—its high concentrations, were confirmed in HF patients with oedema [151]. A well-known endotoxin, it connects with toll-like receptor 4 (TLR-4) and activates an inflammatory cascade due to myocardial disorders such as dysfunction and remodeling of left ventricular and intensified cardiac muscle cells apoptosis [152,153]. GutHeart, a randomized clinical trial, investigates if external interference in intestinal microbiota can improve heart function. Patients are divided into three groups, treated with a placebo, probiotic (Saccharomyces boulardii), or antibiotic (rifaximin). Although the final results of GutHeart are still pending, the first analysis seems to confirm its thesis and indicate a new trend in HF treatment [154]. Recently, Song et al. confirmed a high prevalence of SIBO in HF population, as well as SIBO, was related to worse implications [83].

Trimethylamine-N-oxide (TMAO) is as a pro-atherosclerotic metabolite of phosphatidylcholine [155]. The production of TMAO is dependent on the metabolic pathway of intestinal bacteria, which are responsible for the transformation of phosphatidylcholine into trimethylamine and the latter is oxidized by hepatic enzymes to TMAO [155,156], which has a great impact on atherosclerosis development and increases the risk of chronic vascular disease [140]. No studies prove a direct correlation between TMAO levels and SIBO occurrence, however, Fialho et al. observed TMAO elevation in SIBO-positive patients with NAFLD [53]. Moreover, the results of breath tests of subjects with coronary artery disease (CAD), show a correlation between the prevalence of SIBO and CAD as well as the number of arteries affected by the disease [85]. Interestingly, the elevation of inactive matrix Gla-protein and arterial stiffening was reported in patients with SIBO [84]. Similarly to heart failure, proinflammatory agents are responsible for the induction, development, and exacerbation of CAD [53,84].

Deep vein thrombosis (DVT) may also be associated with SIBO, especially due to the increased concentration of inflammatory factors and expression of TLR-4 by platelets and endothelial cells. Pro-coagulatory activity is induced by combining TLR-4 with LPS; accordingly, SIBO is named as an individual risk factor for DVT [86]. On the other hand, the abundance of cytokines and interleukins in DVT may stimulate the intestinal immune system and alter microbiota in the gut lumen, which indicates DVT as a risk factor for SIBO. A bidirectional relationship between both conditions is highlighted in quoted studies [86,87]. Additionally, the authors advise careful observation of patients with intestinal dysbiosis for findings resembling DVT [86].

#### 4.3.3. Endocrinology

Patients suffering from diabetes mellitus (DM), frequently present with gastrointestinal manifestations like diarrhea, constipation, or flatulence [89,157]. The type of DM is defined by its etiology. Type 1 develops due to the destruction of β-cells in the pancreas, type 2 as a consequence of insulin resistance. After birth gut microbiota plays an important part in the development of the immune system. Firstly, intestinal dysbiosis leads to a dysfunctional immunological response, inducing damage to β-cells. Moreover, higher permeability is also provoked by disturbed bacterial homeostasis in the bowel and the availability of different bacterial antigens which promotes the immune system to destroy β-cells as well. Similarly, in type 2 DM endotoxemia, following the presence of LPS in the bloodstream, induces the production and causes a release of cytokines, which may destroy insulin receptors in the targeted tissue and provoke the onset of type 2 DM [158,159]. These hypotheses could partly explain the role of abnormal microbiota in DM pathogenesis. On the other hand, diabetes itself might influence the intestinal microbiome. It is associated with autonomic neuropathy and acute hyperglycemia, which may provoke disturbances of GI motility, which include delay of gastric emptying and OCTT [89,90,91,92,93,160]. Gastroparesis, as a complication of DM, develops due to the dysfunction of Phase 3 MMC and is often accompanied by SIBO, notably with protracted persistence of symptoms [161]. The prevalence of SIBO in both types of diabetic patients was significantly higher than in the general population. Autonomic neuropathy increases the prevalence of SIBO as well as insulin requirements [88]. In addition, the persistence of dysbiosis could exacerbate diabetes. Interestingly, early results show positive effects of treatment with prebiotics, probiotics, and antibiotics as well as a decrease of GI symptoms in patients with diabetes [162,163].

Merely a few studies about the relationship between the thyroid gland and altered gut microbiome have been published but interesting concepts are being investigated. Endocrine disorders are often accompanied by GI symptoms like abdominal discomfort, constipation, or diarrhea. Hypothyroidism could be a causative factor of hypomotility [164] and due to that SIBO is detected in over 50% of patients with thyroid insufficiency [165]. However, reasons are still vague and different hypotheses like bowel oedema [166] or decreased number of β-adrenergic receptors and myoelectrical function are suggested [94] but also these need more investigations. In one study, massive overgrowth of small intestinal bacteria was considered to influence the neuromuscular system, but the concentration of thyroid hormones was not dependent on the gut microbiota [94]. Surprisingly, Brechmann et al. implied that supplementation of levothyroxine highly interferes with SIBO, even more than hypothyroidism [95]. Hyperthyroidism could influence GI motility as well. Studies propose ghrelin as a causative factor for this, but explanations are still unclear. Differences in the composition of intestinal microbiome between patients suffering from hyperthyroidism and healthy controls have been found, however, results have not established a definite correlation [167]. There are also some suggestions that immune and autoimmune systems could be induced by bacterial antigens and provoke the onset of Graves and Hashimoto disease in genetically susceptible individuals. Dysbiosis modifies the immunological response, which promotes inflammation and leads to vulnerability of the gut wall, increase of intestinal permeability, and endangering of antigens [168]. Both conditions are characterized by the presence of antibodies, which in the first case promote hyperactivity of the thyroid, in the other one the chronic inflammation and destruction of the gland leading to hypothyroidism. Nowadays, good dietary habits play an important role in the improvement of intestinal microbiota, which could lead to a reduction of inflammation. Consumption of optimal amounts of polyunsaturated and omega-3 fatty acids, fiber, or foods rich in secondary plant metabolites (cocoa, honey, fruits, and vegetables) could be beneficial in amelioration of these conditions [169]. Further studies are still required to clarify its correlation with an excess of bacteria in the small intestine [94,95,96].

#### 4.3.4. Neurology

Parkinson’s disease (PD) is the second most common neurodegenerative disorder in the elderly population and besides severe motor symptoms it is also associated with GI motility abnormalities [99,170], which may include small intestine dysbiosis as a result of delayed OCTT. Various studies point to a high prevalence of SIBO in PD and the possibility of it aggravating gastrointestinal manifestations [97,101,102,103,171]. H. pylori co-infection also has a synergic impact on the severity of GI symptoms [101,172]. Contrary, Tan et al. proposed a hypothesis that SIBO-positive patients have a benefit and found a reduced constipation score due to increased GI motility, although SIBO in those patients predicted worse motor function [100]. One of the complications of SIBO-malabsorption could also be caused by levodopa, the mainstay of PD treatment [100,173]. The intestinal mucosa is the absorption area for levodopa and its dysfunction due to microflora abnormalities leads to a decreased concentration of dopamine in the target organ–brain. Moreover, excessive bacterial numbers in the gut might induce the production of reactive oxygen species, which aid in the inactivation of the medication and change its bioavailability. That mechanism was also proposed as a cause of weight loss in PD patients, but it was not confirmed in studies [57]. The prevalence of SIBO is not dependent on PD duration-it can be present in the early stage with the same rate [100]. Therefore, the hypothesis that gut dysbiosis is not only the result but also the cause of PD could be meaningful. Small bowel permeability and endotoxemia activate microglia–brain tissue responsible for neuroinflammation [100]. Furthermore, the intestinal microbiota is considered a potential activator of α-synuclein, a protein inhibitor of dopamine synthesis, and the other “offender” in PD pathogenesis [174]. A hypothesis proposes that improvement of the bacterial status in the gut might have a positive influence on manifestation like also the progress of Parkinson’s disease, but further research is crucial.

Nowadays, autism spectrum disorders (ASDs) are increasingly diagnosed. The pathogenesis of the disease is still unknown. Exacerbating factors are looked for due to differences in course. The severity of autism is divided into three groups (mild, moderate, severe) and it is measured with the Autism Treatment Evaluation Checklist (ATEC) [175]. Additionally, GI manifestations are needed to be assessed with an altered version of the GI Severity Index [176]. A few studies show a significant modification in intestinal bacterial composition. Higher levels of Bacteroidetes or Clostridium perfringens were observed in the autistic population [177]. Contrary, healthy controls presented a greater concentration of Firmicutes [178]. The prevalence of SIBO in autism could even reach above 20% [104]. Wang et al. reported that SIBO was related to the severity of ASD. Children with higher ATEC score presented more intense GI symptoms., which were improved after oral pharmacotherapy with non-absorbable antibiotics [104]. Several pathomechanisms are mentioned as likely causes of these relationships. Firstly, propionate overproduction by Clostridia species could lead to neurological impact as well as modified bacterial metabolism of aromatic acids [179]. Secondly, malabsorption, including vitamin B12 malabsorption is observed in the course of SIBO [180]. Persistent cobalamin deficiency may yield neurological and psychiatric disorders by glutathione deficiency as a result of higher antioxidant take-up [181]. Finally, LPS of Gram-negative bacteria leads to higher permeability of the blood-brain barrier by inducing inflammatory status in the brain [182]. It could generate toxins (like mercury), responsible for aggravating the course of autism, in the central nervous system. Unfortunately, most research was performed on animals and additional studies are necessary to confirm those hypotheses on humans. However, a confirmed relationship between SIBO and ASD may create new treatment possibilities in the autistic population that would improve their quality of life.

Brain Fogginess (BF)–a rare neurological condition, also known as D-lactate encephalopathy, is induced by elevated intestinal production and absorption of D-lactate due to D-lactic acidosis. Neurological BF manifestations, like confusion, slurred speech, ataxia, and impaired short-term memory are accompanied by abdominal pain, belching, or flatulence, though initially, it was associated only with short bowel syndrome. The main cause is the abundance of Lactobacillus and Bifidobacterium species, which are responsible for the fermentation of carbohydrate substrates due to excessive production of D-lactate [105,183]. Furthermore, the mentioned disturbances were observed not only in patients with short bowel syndrome. Rao et al. described the possible relation of BF to gut dysbiosis. In their research, the occurrence of SIBO and D-lactic acidosis was significantly higher in subjects with BF than in those without BF. However, further investigations are essential due to many different limitations of the study [105].

#### 4.3.5. Rheumatology and Cutaneous Diseases

Systemic sclerosis (SSc, scleroderma) is a chronic, connective tissue disorder of autoimmune etiology, which leads to fibrosis of numerous organs [109,184]. GI tract, starting with the esophagus and following with the large and small intestine, may be afflicted by a pathological process in up to 90% of patients [185,186,187]. Approximately 55% of patients suffer from gastrointestinal symptoms, like bloating, constipation, abdominal pain, or diarrhea [106,109,188], which substantially influences their quality of life [106]. Dysmotility, which manifests as prolonged OCTT and impaired bowel clearance, could be a risk factor for the development of SIBO [106,109]. The co-occurrence of SSc and SIBO was confirmed in various studies [106,108,110,111,112,113,114]. Disease duration of longer than 5 years is a significant risk factor for SIBO development [109]. Mortality in scleroderma is influenced by one of the major complications of gut bacteria excess–malnutrition [185,187], hence it is proposed that SIBO should be diagnosed and cured at an early stage [112]. Lower levels of protein, vitamin B12, and ferritin were observed in SIBO-positive subjects contrary to SIBO-negatives [107]. Jejunal bacteria are responsible for the deconjugation of bile salts and insufficient absorption of fat and fat-soluble vitamins like A or K [189]. Screening is suggested, when the global symptoms score (GSS) of digestive symptoms is greater than 5 [107]. The GSS incorporates abdominal pain, vomiting, bloating, or diarrhea [190]. Antibiotics, especially rifaximin 1200 mg daily, are commonly used during eradication, mainly because of their safety and efficacy. Reduction of gastrointestinal symptoms was observed after completion of therapy in systemic sclerosis patients [106,188]. Additionally, as evidenced by recent studies, probiotics like Saccharomyces boulardii may be effective in the reduction of manifestations from the GI tract, sometimes even in combination with metronidazole [114]. However, more research is still required to routinely treat all patients in that manner [191].

Although rosacea is classified as a dermatosis, manifesting with erythema and phymatous changes of the facial area [192], patients frequently complain of gastrointestinal disorders as well [193]. Rosacea subjects demonstrate the occurrence of SIBO more frequently than control groups. Furthermore, disturbances of the small gut microbiota were confirmed as a potential trigger of rosacea. The proposed pathogenesis seems to be the presence of bacterial antigens in the bloodstream and the production of tumor necrosis factor α (TNF-α). Interestingly, treatment with rifaximin can achieve a reduction or remission of dermatosis in SIBO-positive patients, which confirms a relationship between bowel microbiome and rosacea [115,116]. More studies are still needed to elucidate the mechanisms of the aforementioned processes.

#### 4.3.6. Nephrology

Gut-kidney axis is a novel research area undertaken by dozens of scientists. All current findings point out a huge impact of intestinal milieu on renal functionality. In chronic kidney disease (CKD) the majority of patients suffer from GI manifestation. Kidney failure predisposes to changes in the intestinal microbiome as well as in the gastroenterointestinal system. Overgrowth of aerobic and proteolytic bacteria in the duodenum and jejunum is observed in the CKD population. Expansion of Enterobacteriaceae and Enterococci species with a relative deficit of Lactobacillus, Prevotella, or Bifidobacterium seems to be meaningful [194]. Moreover, modifications in the microscopic structure of the lumen (enlargement of the crypts, decrease of villous, penetration of lamina propria with inflammatory cells, disarrangement of epithelial tight junction) are present in CKD [195]. Solutes, generated by bacteria (such as indoxyl sulfate or p-cresol sulfate), are normally eliminated with urine, consequently, this process is disturbed in CKD [196]. The prevalence of SIBO reaches up to 36% and it is significantly higher than in healthy controls [117]. Uremia is proposed to be the culprit of this complication. Uremic toxins act on the autonomic nervous system and establish conditions for bacterial growth by aggravating intestinal motility. On the other hand, CKD contributes to a rise in intestinal permeability and penetration of antigens into the bloodstream [197]. Toxins may be intercepted by anion transporters in the tubules, which leads to the deterioration of the kidney [198]. This could be one explanation for the influence of intestinal dysbiosis on the progression of CKD.

Acute kidney injury (AKI) is another disease where studies show that lack of exposure to normal microbiota induces less immune deviation and reduces immune regulation [199]. Ischemia-reperfusion injury (IRI), as the leading trigger of AKI [200], could induce intestinal dysbiosis and exacerbate kidney injury by boosting inflammation consequently. Yang et al. discovered that the reduction of gut microbiota in mice may have renoprotective qualities by depletion of Th17, Th1 response and progress of Tregs, and M2-polarized macrophage [201]. Additionally, intestinal microbiota by secretion of SCFAs, which diminish inflammation in the kidney, could control systemic implications of AKI [202].

Immunoglobulin A (IgA) is abundant in the intestinal tract. Chronic pro-inflammatory stage and an excessive number of gut microbiota boost epithelium and lymphocytes B to IgA production. Therefore, intestinal dysbiosis is considered a risk factor for the development of IgA nephropathy [203]. Genome-wide association studies have identified risk loci in genes involved in the maintenance of the intestinal epithelial barrier and response to mucosal pathogens. The genetic risk of IgA nephropathy also strongly correlates with microbiota variation, particularly helminth diversity [204].

The kidney-gut axis seems to also play a role in hypertension development. Studies have shown that both in animal models and a small sample of patients there is a decrease in the microbial abundance, diversity, and an increased Firmicutes/Bacteroidetes ratio. A microbiome-oriented intervention (minocycline) reduced blood pressure values [205]. Another paper revealed a relationship between gut dysbiosis and blood pressure in obstructive sleep apnea-induced hypertension [206].

A small study also suggests that all kidney stone formers have a distinct gut microbiome (both Bacteroides and Prevotella abundance were associated with nephrolithiasis) [207]. However, data on the direct relationship between kidney diseases and SIBO is still lacking, and detailed research is crucial so that it could be applied in future diagnosis and treatment.

## 5. Conclusions

**Figure 2:**
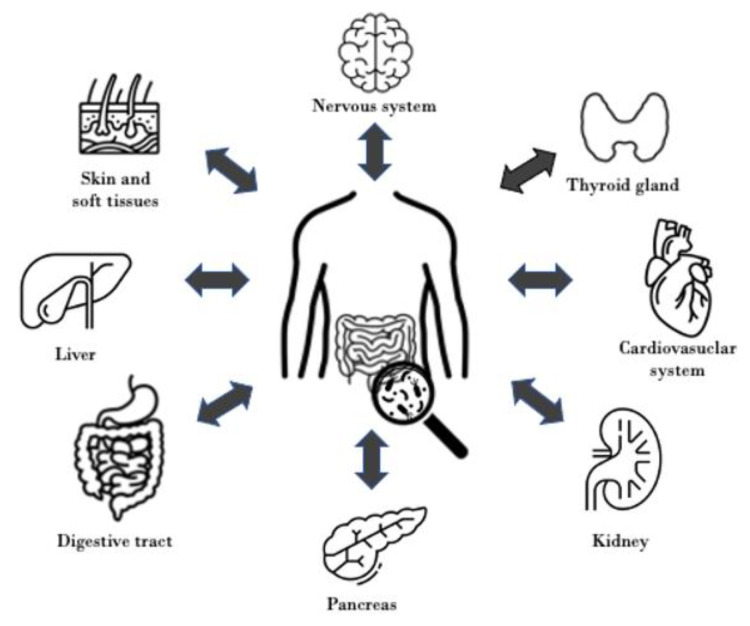
Bidirectional relationships between altered intestinal microbiota and different regions of the human body.

In conclusion, SIBO is a frequent condition, and many factors could promote its development. Although there are different types of diagnostic tests, none is perfect, that’s why new diagnostic strategies are being sought. Bacterial dysbiosis is observed not only in patients with gastrointestinal symptoms but also related to other systemic diseases. Multiple studies confirm a constellation of correlations between digestive, cardiovascular, endocrine, neurological, nephrological, connective tissue, or dermatological disorders and SIBO confirming its multifactorial impact on various medical problems (Figure 2). Some of them are more complex, others just a trifle. They all show the prominent impact that gut microbiota has on our organism. However, additional research is crucial to verify many faces of SIBO. Antibiotic therapy could reduce not only GI symptoms but also manifestations of underlying pathology, hence the quality of life could be improved. This creates new treatment directions for many well-known diseases. However, the biggest problem of most studies cited is little number and diversity of tested groups and the requirement of verification in other similar studies. Moreover, a lot of findings are based on animal models and have not been confirmed in humans. The other problem is the disparity in diagnostic methods as well as the variety of SIBO manifestations. It makes it impossible to compare studies’ results with each other. Finally, a lot of conditions described in this paper were connected only with gut microbiota status but not directly with SIBO. While these studies may have some limitations, they still provide valuable insights and have the potential to impact future research in this area. These studies can serve as a foundation for further investigation and can help to identify new directions for research. Despite their limitations, these studies highlight the complexity of SIBO and the need for continued investigation into this important area of study.

## Figures and Tables

**Table 1:** Original studies investigating the co-existence of SIBO with different disorders.

Disorder	Years of Publications		Number of Studies	Authors, References
Gastroenterology				
Helicobacter pylori infection	2017		1	Enko et al. [6]
Irritable bowel syndrome	1991–2016		14	Pimental et al. [7], Pimental et al. [8], Walters et al. [9], Lupascu et al. [10], Posserud et al. [11], Bratten et al. [12], Park et al. [13], Parodi et al. [14], Ghoshal et al. [15], Rana et al. [16], Sachdeva et al. [17], Ghoshal et al. [18], Abbasi et al. [19], Zhao et al. [20]
Inflammatory bowel disease	Crohn’s disease	1981–2018	5	Rutgeerts et al. [21], Castiglione et al. [22], Klaus et al. [23], Greco et al. [24], Ricci et al. [25]
	Ulcerative colitis	2014, 2021	2	Rana et al. [26], Yang et al. [27]
	Both	2013–2016, 2022	4	Rana et al. [28], Lee et al. [29], Andrei et al. [30], Ghoshal et al. [31]
Celiac disease	1970; 2002–2015		7	Prizont et al. [32], Tursi et al. [33], Ghoshal et al. [34], Rana et al. [35], Rubio-Tapia et al. [36], Chang et al. [37], Lasa et al. [38]
Abdominal surgery	2011–2020		6	Paik et al. [39], Heneghan et al. [40], Sabate et al. [41], Kim et al. [42], Rao et al. [43], Mouillot et al. [44]
Obesity	2008–2018		4	Sabate et al. [45], Madrid et al. [46], Fialho et al. [47], Roland et al. [48]
Non-alcoholic fatty liver disease	2001–2017		8	Wigg et al. [49], Sajjad et al. [50], Miele et al. [51], Shanab et al. [52], Fialho et al. [53], Ghoshal et al. [54], Mikolasevic et al. [55], Shi et al. [56]
Cirrhosis	1991–2016		11	Chesta et al. [57], Casafont Morencos et al. [58], Madrid et al. [59], Yang et al. [60], Bauer et al. [61], Gunnarsdottir et al. [62], Nancey et al. [63], Pande et al. [64], Jun et al. [65], Gupta et al. [66], Zhang et al. [67]
Gall stone disease	2014, 2018		2	Kaur et al. [68], Kim et al. [69]
Chronic pancreatitis	1985–2019		8	Casellas et al. [70], Trespi et al. [71], Signoretti et al. [72], Kumar et al. [73], Kim et al. [74], Ni Chonchubhair et al. [75], Lee et al. [76]
Acute pancreatitis	2017, 2020		2	Zhang et al. [77], Kim et al. [78]
Cystic fibrosis	2009–2019		3	Lisowska et al. [79], Dorsey et al. [80]., Furnari et al. [81]
Cardiology				
Heart failure	2016, 2021		2	Pasini et al. [82], Song et al. [83]
Atherosclerosis	2017, 2018		2	Ponziani et al. [84], Fialho et al. [85]
Deep vein thrombosis	2016, 2017		2	Fialho et al. [86], Cheng et al. [87]
Endocrinology				
Diabetes	Type 1	2009, 2013, 2018	3	Ojetti et al. [88], Faria et al. [89], Malik et al. [90]
	Type 2	2011, 2017	2	Rana et al. [91], Rana et al. [92]
	Both	2000, 2002	1	Zietz et al. [93]
Thyroid disorders	2007, 2017, 2018		3	Lauritano et al. [94], Brechmann et al. [95], Konrad et al. [96]
Neurology				
Parkinson disease	1996–2020		7	Gabrielli et al. [97], Dobbs et al. [98], Fasano et al. [99], Tan et al. [100], Niu et al. [101], Su et al. [102], Hasuike et al. [103]
Autism	2018		1	Wang et al. [104]
Brain fogginess	2018		1	Rao et al. [105]
Rheumatology and cutaneous disaeses				
Systemic sclerosis	1980–2020		9	Parodi et al. [106], Marie et al. [107], Fynne et al. [108], Savarino et al. [109], Gemignani et al. [110], Marie et al. [111], Adarsh et al. [112], Sawadpanich et al. [113], Garcia-Collinot et al. [114]
Rosacea	2008, 2016		2	Parodi et al. [115], Drago et al. [116]
Nephrology				
Chronic kidney disease	2003		1	Strid et al. [117]

## Data Availability

No new data were created or analyzed in this study. Data sharing is not applicable in this article.
